# Label-Free and Bioluminescence-Based Nano-Biosensor for ATP Detection

**DOI:** 10.3390/bios12110918

**Published:** 2022-10-24

**Authors:** Elham Karimi, Maryam Nikkhah, Saman Hosseinkhani

**Affiliations:** Department of Nanobiotechnology, Faculty of Biological Science, Tarbiat Modares University, Tehran 14115-175, Iran

**Keywords:** bioluminescence, firefly luciferase, plasmonic metal nanoparticles, ATP assay

## Abstract

A bioluminescence-based assay for ATP can measure cell viability. Higher ATP concentration indicates a higher number of living cells. Thus, it is necessary to design an ATP sensor that is low-cost and easy to use. Gold nanoparticles provide excellent biocompatibility for enzyme immobilization. We investigated the effect of luciferase proximity with citrate-coated gold, silver, and gold–silver core–shell nanoparticles, gold nanorods, and BSA–Au nanoclusters. The effect of metal nanoparticles on the activity of luciferases was recorded by the luminescence assay, which was 3–5 times higher than free enzyme. The results showed that the signal stability in presence of nanoparticles improved and was reliable up to 6 h for analytes measurements. It has been suggested that energy is mutually transferred from luciferase bioluminescence spectra to metal nanoparticle surface plasmons. In addition, we herein report the 27-base DNA aptamer for adenosine-5′-triphosphate (ATP) as a suitable probe for the ATP biosensor based on firefly luciferase activity and AuNPs. Due to ATP application in the firefly luciferase reaction, the increase in luciferase activity and improved detection limits may indicate more stability or accessibility of ATP in the presence of nanoparticles. The bioluminescence intensity increased with the ATP concentration up to 600 µM with a detection limit of 5 µM for ATP.

## 1. Introduction

The phenomenon of light emission in living organisms is known as bioluminescence [1,2]. Bioluminescence proteins, such as luciferases, are among the most sensitive probes for gene expression detection [3,4], ATP assay and drug screening [1,5,6], and contamination detection in ecosystems [7,8]. The firefly luciferase requires ATP and O_2_ to catalyze the two-step oxidation of luciferin to produce light (maximum emission at 560 nm) [9]. Furthermore, the *Renilla* luciferase is a 38 kDa protein that generates light upon simply adding coelenterazine (CTZ) (maximum emission at 480 nm) [10]. Despite the numerous applications and benefits of luciferase, the poor signaling intensity and short reaction half-life have prevented its widespread adoption [6,11,12]. There are many ways to stabilize the enzyme and alter its activity and emission spectra, such as immobilization on metal nanoparticles and quantum dots or bioluminescence resonance energy transfer (BRET). However, BRET probes such as luciferase-conjugated quantum dots (BRET-QDs) have been examined due to their cadmium content, which is known to be toxic [13,14]. The surface plasmon resonance (SPR) is exceptionally narrow and powerful in metal nanoparticles such as gold and silver. The wavelength of the SPR peak and the scattering efficiency of the nanoparticles are the most critical factors that determine the enhancement/quenching influence on fluorophore molecules [15,16].

Due to their high surface energy, metal nanoparticles interact with biomolecules such as proteins, lipids, and nucleic acids. Protein interaction with ions and bulk materials can result in structural damage and activity loss. Enzyme immobilization on novel nanoparticles, on the other hand, may alter their catalytic activity, structure, and stability [17]. Chemical reactions in bioluminescence produce electronically excited states, emitting light without an external excitation [18]. The interaction of the SPR peak of metal nanoparticles with the excited state of bioluminescent molecular species may result in photoluminescence enhancement or quenching, depending on the distance between the emitter and the metal nanoparticles and changes in the excited state lifetime [5]. However, despite many reports on BRET using nanoparticles, a few bioluminescence enhancements have been described by metal nanoparticles within *E. coli* in the presence of silver [11] and gold nanoparticles [19,20]. For bioactive discovery, a whole-cell bioluminescent biosensor in silver nanoparticles was tested, which showed enhanced bioluminescence and metal-enhanced fluorescence [21]. In another study, Ebrahimi et al. (2015) showed that *P. pyralis* luciferase could be electrostatically immobilized on functionalized magnetic nanoparticles (MNPs) of His-tag with Ni^2+^/Cu^2+^ ion immobilization, which decreased the affinity for ATP while increasing the affinity for luciferin without affecting thermal stability or longevity [22]. The effect of gold nanoparticles on the purified recombinant green-emitting click beetle luciferase and red-emitting railroad worm luciferase has also been reported [23]. The fluorescent metal nanoclusters have tens of atoms and behave like molecules without a featured surface plasmon resonance peak in the visible region. Nevertheless, they are fluorescent in the visual to the near-infrared range [24,25]. It is known that gold nanoparticles with sizes of more than 3 nm show the LSPR absorption peak (~520 nm) in the UV/visible spectrum, while gold nanoclusters with sizes less than 2 nm display no LSPR peak [26]. Therefore, the association of these luciferases with metal nanoparticles and nanoclusters is a promising strategy to increase their stability further and maintain their luminescence for longer times for some bioanalytical and bioimaging applications. Firstly, we investigated the possibility of improved bioluminescence in the presence of metal nanoparticles such as gold, silver, and gold–silver nanoparticles, gold nanorods, and BSA–Au nanoclusters.

For the first time, we compare the effect of a variety of nanoparticles with the plasmonic feature, including gold nanorods with visible and infrared absorption spectra and luciferases with a variety of emission spectra, including mutant luciferases (red emitter). This study aims to improve the kinetic and structural properties of firefly luciferase, including enzyme activity and stability. Then, we used gold nanoparticles and ATP aptamer for bioluminescent-based ATP assay. ATP assays are procedures that can measure cell viability on the basis of the detection of ATP. All living cells, including bacteria, can be detected using ATP assays. Therefore, the varying ATP concentration can indicate several diseases, such as malignant tumors, hypoglycemia, and Parkinson’s disease [27,28]. Several detection methods, such as colorimetric, fluorescent, and bioluminescent, can be used. Most researchers choose bioluminescent ATP assays to measure cell viability due to higher sensitivity, simple and homogeneous protocol, and fast results. Thus, it is necessary to design an ATP sensor that is low-cost and easy to use. Aptasensors for ATP detection have recently attained much attention due to their superior biocompatibility, high specificity, and accessible synthesis [29,30].

## 2. Materials and Methods

### 2.1. Reagents

HAuCl_4_·3H_2_O (99.9%), NaBH_4_ (99%), ascorbic acid (99%), hexadecyl trimethyl ammonium bromide (CTAB) (99%), ascorbic acid (99%), AgNO_3_ (99%), and bovine serum albumin (BSA) were purchased from Sigma Aldirich (St. Louis, MO, USA). Lactose and trisodium citrate were purchased from Merck (Darmstadt, Germany), Isopropyl d-thiogalactopyranoside (IPTG) Takara (Shiga, Japan), ATP from Roche (Basel, Switzerland), coelenterazine and D-luciferin potassium salt from Resem (Lijnden, Netherlands). The Ni-NTA spin kit was provided from Qiagen Inc (Hilden, Germany). Deionized water (Millipore Milli-Q grade) with a resistivity of 18.2 MΩ·cm was used in all experiments. Glassware was thoroughly cleaned with aqua regia and rinsed with DI water. 

### 2.2. Expression and Purification of Luciferase Enzymes 

Five milliliters of Luria–Bertani (LB) medium containing 50 and 100 mg·mL^−1^ kanamycin and ampicillin, respectively, were inoculated with a new bacterial colony harboring the expression plasmid and grown at 37 °C overnight with 180 rpm shaking.

Then, 200 mL of 2XYT medium with 500 µL overnight culture was inoculated and grown at 37 °C with 180 rpm shaking until the OD_600_ reached 0.6, followed by the addition of IPTG and lactose to the 2XYT medium at final concentrations of 0.5 mM and 4 mM, respectively, and then the mixture was incubated at 20 °C overnight with 180 rpm shaking. We centrifuged the cells at 5000 rpm for 5 min and then treated them with the lysis buffer (50 mM Tris-HCl, 300 mM NaCl, and 10 mM imidazole (pH 7.8)). The His6-tagged fusion protein was purified using the manufacturer’s Ni-NTA spin column (Qiagen).

### 2.3. Preparation of Gold Nanoparticles (Au NPs)

The Turkevich method [31] was used to create 10 to 30 nm diameters of gold nanoparticles. In brief, 100 mL of Milli-Q water was refluxed in a three-necked flask, mixed with 400 µL of HAuCl_4_ (250 mM), and allowed to boil before adding 1 mL of trisodium citrate (38.8 mM) solution and heating for 15 min. After creating a ruby red color, the temperature and stirrer were turned off and stored at 4 °C until other use.

### 2.4. Preparation of Silver Nanoparticles (Ag NPs)

We synthesized silver nanoparticles as described by Chen et al. [32] with brief changes. AgNO_3_ aqueous solution (0.5 mL, 10 mM) was added to ultrapure water (10 mL) and, after boiling, trisodium citrate (0.3 mL, 38.8 mM) solution and sodium borohydride solution (0.1 mL, 10 mM) were added to solution immediately with stirring. The formation of the Ag nanoparticles was confirmed when the solution color changed to yellow.

### 2.5. Preparation of Core–Shell Au–Ag Nanoparticles

The method described by Xia et al. [33] was used to create spherical core–shell Au–Ag nanoparticles. In brief, 0.5 mL of HAuCl_4_ aqueous solution (10 mM) and AgNO_3_ solution (10 mM) were added to 5 mL of boiling water while constantly stirring under reflux. Subsequently, trisodium citrate (0.2 mL, 38.8 mM) and sodium borohydride aqueous solution (0.8 mL, 10 mM) were added. Within 3 min, the reaction mixture turned in black, then orange, and finally ruby red. The reaction was allowed to run for 30 min to form uniform spherical nanoparticles before being stored at 4 °C until further use.

### 2.6. Preparation of BSA–Au Nanoclusters (BSA-AuNCs)

Xie et al. [34] developed a method for synthesis of gold nanoclusters that was used in this study. All glassware was washed in Aqua Regia (HCl/HNO_3_ volume ratio of 3:1) and rinsed in ultrapure water. In a typical experiment, 1 mL of aqueous HAuCl_4_ solution (10 mM) was added to 1 mL of BSA solution (20 mg·mL^−1^) while vigorously stirring. After 10 min, a 0.1 mL (1M) NaOH solution was added to raise the pH above 12. The reaction solution was continuously shaken at 37 °C for 12 h. After synthesizing the BSA-based nanocluster, the final solution was light brown. Additionally, the red fluorescence emission suggested the formation of AuNCs.

### 2.7. Preparation of Gold Nanorods (GNRs)

As previously described, we synthesized the gold nanorods using a sequential seed-mediated growth method [35,36]. In brief, the seed solution was made by combining 250 µL of 0.01 M gold salt (HAuCl_4_) aqueous solution with 7.5 mL of 0.095 M surfactant (CTAB); immediately, 0.6 mL of the ice-cold solution of 0.01 M NaBH_4_ was added, and the reactants were mixed by fast inversion for 2 min, yielding a pale yellow-brown solution. After that, 400 µL of 0.01 M HAuCl_4_, 9.5 mL of 0.095 M cetyltrimethylammonium bromide (CTAB, as a template), 64 µL of 0.10 M ascorbic acid (as a reducing agent), and 60 µL of 0.01 M AgNO_3_ were added to the seed solution (for shape induction). Finally, 40 µL of seed particles were added to the container gently shaken for 10 s to allow GNRs to grow.

### 2.8. Immobilization of Enzyme with Metal-NPs

The metal NPs were dissolved in Tris-HCl buffer (50 mM, pH 7.8) at 4 °C with the determined concentration of luciferase. The concentration of metal nanoparticles was varied while keeping the concentration of luciferases constant to achieve the best concentration of nanoparticles that improved luciferase activity.

### 2.9. Luciferase Activity Assay

We studied the effect of metal nanoparticles on luciferase luminescence in vitro by expressing and purifying the firefly and *Renilla* and mutant firefly luciferase in *E. coli* BL21 (DE3). 

### 2.10. UV/Visible Spectra

The maximum absorbance peak of metal nanoparticles was measured at room temperature and in a 1 cm quartz cuvette using a PerkinElmer UV/Visible spectrophotometer Lambda 365 at room temperature.

### 2.11. Particle Size Distribution and Zeta Potential

The size distribution and zeta potential of metal nanoparticles were measured with a Malvern zeta sizer nanosize device outfitted with a HeNe laser source (ƛ=633).

### 2.12. Transmission Electron Microscopy (TEM)

TEM was used to investigate the size and morphology of metal nanoparticles (Philips CM 200, 200 kV TEM, ATM 2 k × 2 k CCD camera).

### 2.13. Fluorescence Spectra Measurement 

Photoluminescence (PL) spectra were obtained on an RF6000 Shimadzu fluorescence spectrophotometer and outfitted with a 150 W Xenon lamp and a slot width of 5 nm, as well as a 1 cm quartz cell. Following excitation at 285 nm, the fluorescence spectra of Fluc (firefly luciferase), Rluc (*Renilla* luciferase), and Mfluc (mutant-firefly luciferase) treated with metal nanoparticles were measured directly after mixing in the range 280–400.

### 2.14. Circular Dichroism (CD) Analysis

Circular dichroism (CD) spectra were recorded on Jasco J-715 spectrophotometer at 25 °C. The far CD spectra were taken at a protein concentration of 0.2 mg mL^−1^ in 50 mM Tris-HCl buffer (pH 7.8) using a 1 mm path in the presence and absence of the metal nanoparticles. All spectra were collected from 200 to 250 nm, and the background was replaced against buffer blank. 

### 2.15. Bioluminescence Spectra (BL Spectra) 

BL spectra were recorded using a Cytation^TM^ 3 (BioTek) from 300 to 700 nm. The emission spectra of the purified firefly luciferase, mutant firefly luciferase, and *Renilla* luciferase were obvious after adding luciferin (and Mg^2+^-ATP), and coelenterazine. Bioluminescence spectra were recorded in the presence and absence of nanoparticles to study the effect of nanoparticles on the intensity of light emission. 

### 2.16. AuNP Stability Test

We designed a bioluminescence-based aptasensor for ATP sensing. For this purpose, we evaluated the effect of targets on AuNP stability; then, 8 μL of 1 M NaCl was mixed with the same volume of synthesized AuNPs (13 nm, ∼0.5 nM), ATP–AuNP binary complex (2 mM ATP), and ATP/aptamer/AuNP ternary complex (2 mM ATP, 5 µM aptamer) titrated in a 96-well plate. After each addition, their absorption spectra were recorded with Cytation^TM^ 3 (BioTek). The ATP aptamer was ACCTGGGGGAGTATTGCGGAGGAAGGT. 

### 2.17. Adenosine Triphosphate (ATP) Assay

The gold nanoparticles with a concentration of 0.5 nM and aptamer with a concentration of 5 μM were mixed at room temperature. Then, different concentrations of ATP (0.001–0.6 mM) were added to gold nanoparticles, aptamer, and the combination of nanoparticles and aptamer. Enzyme activity (enzyme concentration was 0.8 μM) at different ATP concentrations was recorded by a luminometer.

### 2.18. Cell Culture

The human breast carcinoma MCF-7 cells were cultured in DMEM medium, supplemented with 10% fetal bovine serum (FBS), and 1% antibiotics (penicillin/streptomycin). Then, the cell culture was incubated at 37 °C under a humidified atmosphere containing 5% CO_2_. At different concentrations, we employed doxorubicin (Dox), a well-known anthracycline-derived chemotherapeutics in apoptosis [37]. To this purpose, MCF-7 cells (3 × 10^5^ cell/mL) were seeded in each well of a 24-well plate overnight; then, the cells were incubated with Dox at various concentrations (100–500 ng·mL^−1^ in DMEM). After 24 h incubation, the cells were harvested and rinsed with PBS buffer. Three replicates were performed for each treatment.

## 3. Results

In order to see the plasmonic effect on bioluminescence properties of luciferases, citrate-coated Au NPs, Ag NPs, Au–Ag NPs, and GNRs (gold nano rods) were synthesized and characterized.

### 3.1. Nanoparticle Characterization

**Figure 1:**
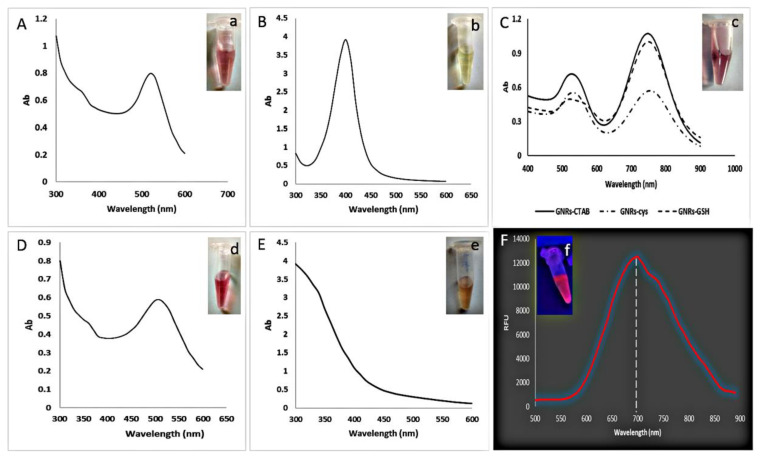
UV/visible absorption spectra of (**A**) Au NPs, (**B**) Ag NPs, (**C**) GNRs, (**D**) Au–Ag NPs, (**E**) BSA-AuNCs, and (**F**) fluorescence spectra of BSA-AuNCs (inset images; (**a**–**e**) under room light; (**f**) light emission under UV light). RFU: relative fluorescence units, Ab: absorbance.

TEM analysis and UV/visible spectroscopy were used to analyze the metal nanoparticles. Figure 1A–F shows the UV/Vis spectra of metal NPs in the 200–600 nm region of spectra. In this case, the local surface plasmon resonance (LSPR) capabilities are greatly dependent on the material, size, and form of the utilized metallic nanoparticles [38]. Noble metal nanoparticles exhibit distinct adsorption in the visible area of the electromagnetic spectrum, which is associated with their collective oscillation of free electrons. Synthesis of Au NPs, Ag NPs, GNRs, Au–Ag core–shell, and BSA-NCs were verified by their absorption peak at 525, 400, 520–750, 500, and 350 nm, respectively (Figure 1A–E). The citrate-capped Au NPs and Au–Ag NPs were produced as ruby red (Figure 1a–d), and the change in color from white to yellow demonstrated the production of the silver nanoparticles (Figure 1b). BSA is a well-characterized protein that has long applications as a capping agent for the formation of nanoclusters due to its high affinity for inorganic salts [39]. In this study, we synthesized a BSA–Au nanocluster that emitted a highly fluorescent light with a maximum wavelength at 700 nm (Figure 1F,f).

In our work, synthesis of GNRs was performed utilizing the seed-mediated growth technique in the presence of CTAB as a surfactant. The seed solution was generated by reducing HAuCl_4_ with ice-cold NaBH_4_ in the presence of the CTAB surfactant. The growth solution was made by dissolving HAuCl_4_ in a specified volume of AgNO_3_ solution in the presence of CTAB and then addition of ascorbic acid. Then, the GNRs began to grow immediately after the seed solution was added to the growth solution. The CTAB surfactant is required to synthesize GNRs because it acts as a “structure-directing agent” to control the final particle shape and as a stabilizer to prevent GNRs from aggregation during the synthesis [36,40]. The synthesized GNRs exhibited two absorption peaks within 520–750 nm and were ruby red (Figure 1C,c). Typically, the GNR surface is modified by substituting thiol-terminated ligands for the connected CTAB bilayers, which are then firmly bonded to the GNR surface via the strong Au–S covalent interactions. We changed the surface of GNRs in our work using cysteine and glutathione because these compounds contain free thiol that can form a strong binding with the surface of GNRs. After cysteine and glutathione binding, the absorption spectra of gold nanorods, showed a drop in intensity and a shift of the higher absorption peak in both visible and infrared ranges (Figure 1C).

**Figure 2:**
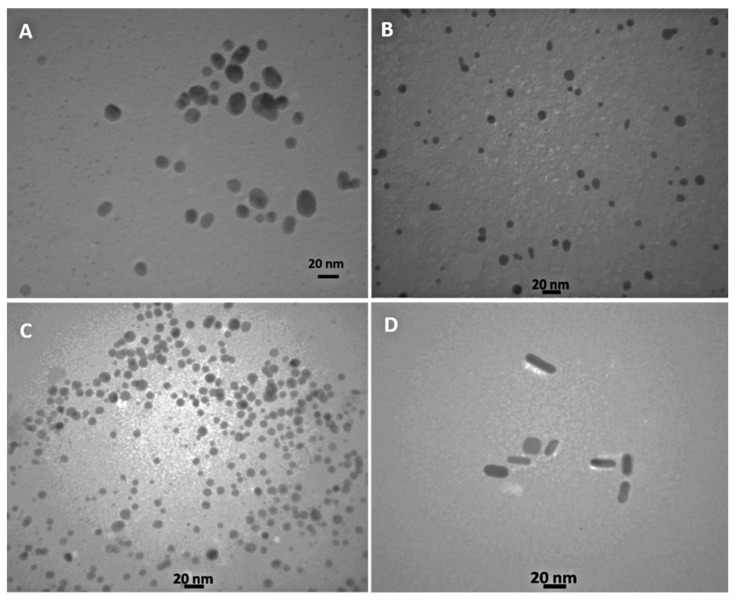
TEM images of (**A**) Au NPs, (**B**) Au–Ag NPs, (**C**) Ag NPs, and (**D**) GNRs.

Transmission electron microscopy (TEM) was used to examine the structure and uniformity of the synthesized metal nanoparticles. Figure 2A–D shows the TEM micrograph of Au NPs, Au-Ag NPs, Ag NPs, and GNRs, respectively, which were well dispersed and were approximately spherical for metal nanoparticles and rod-shaped for GNRs.

### 3.2. Nanoparticle Effect on Luciferase Activity

**Figure 3:**
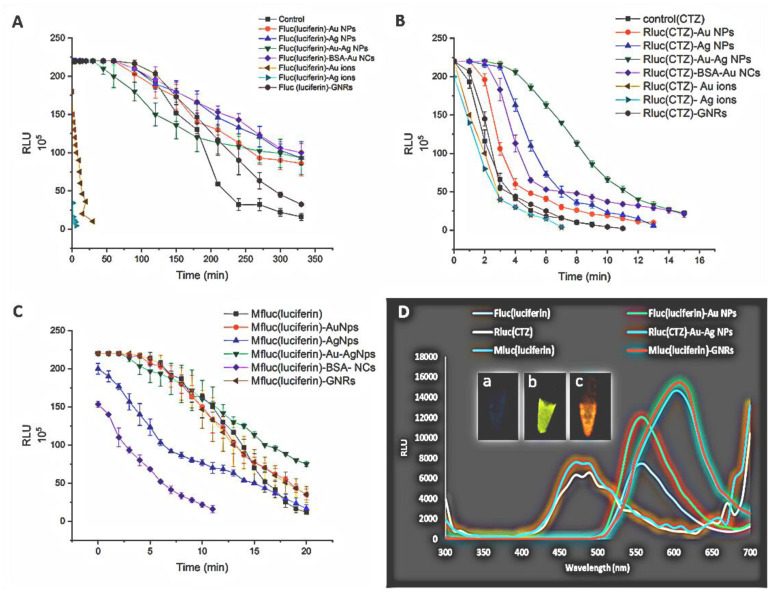
(**A**–**C**) Influence of different metal nanoparticles and metal ions on the activity of luciferase enzyme; (**D**) bioluminescence emission spectra of free luciferase and bound luciferase metal NPs at 25 °C, pH 7.8 (inset images (**a**–**c**) in vitro bioluminescence imaging of Fluc, Rluc, and Mfluc). RLU: relative light units.

Figure 3A–C shows the effect of metal nanoparticles on the luciferase activity. We also monitored the enzyme activity after adding the substrate. The results showed that all metal nanoparticles had an activator effect on the firefly luciferase; however, the impact of silver nanoparticles and BSA nanoclusters was more significant. The BSA protein alone can stabilize enzymes, and we presume that the enhancing effect of BSA-NCs may be due to itself because the BSA–Au nanoclusters have absorbance at 350 nm, which does not overlap with the maximum emission of firefly luciferase at 560 nm. As a demonstration, Figure 3A presents that, after the addition of substrate and incubation at room temperature for 6 h, native Fluc lost more than 90% of its original activity, while complexes of nanoparticles of gold, silver, gold–silver core–shell, and nanoclusters increased the enzyme activity approximately fivefold after 5 h. Furthermore, the enzymatic activity of Ag and Au ions was severely decreased in less than 1 h.

On the other hand, *Renilla* luciferase activity was increased 3–5 times more than free enzyme in the presence of gold, silver, gold–silver core–shell nanoparticles, and BSA–Au nanoclusters until 15 min. In the presence of gold nanorods, an increase in luciferase activity was observed only within 10 min. In addition, *Renilla* luciferase activity reached to less than 1% in 5 min in the presence of metal ions. Gold–silver core–shell nanoparticles had the most excellent outcome, with the SPR spectrum (λ_max_ = 500 nm) overlapping well with the maximum emission of *Renilla* luciferase at 480 nm (Figure 3B).

Gold nanorods, gold, and gold–silver core–shell nanoparticles increased mutant firefly luciferase activity up to three times compared to free enzyme after 20 min, but Mfluc activity in the presence of silver nanoparticles and BSA–Au nanoclusters was drastically reduced to 10% in 10 min gold–silver nanoparticles (λ_max_ = 500 nm) and, to a lesser extent, gold nanoparticles (λ_max_ = 520 nm) and gold nanorods (λ_max_ = 520–750 nm) showed an increasing effect on the mutant enzyme with the maximum emission in the red region (λ_max_ 620 nm). We suppose that gold nanorods with two absorption peaks at 520 and 750 nm overlapped well with the enzyme emission and showed a greater effect on the activity of the native and red-emitter mutant of firefly luciferase. In contrast, we did not find significant variation in the activity of the *Renilla* luciferase (Figure 3C).

It may be suggested that the increase in firefly, *Renilla*, and mutant (red-emitter) luciferase activities in the presence of metal nanoparticles is due to the overlapping of enzyme emission spectra with SPR peak of metal nanoparticles, as well as coupling of emission energy due to enzyme activity and surface plasmon resonance of nanoparticles. The emission spectrum of the red-emitter enzyme (λ_max_ = 620 nm) and the SPR peak of silver nanoparticles (λ_max_ = 400 nm) slightly overlap. In contrast, the enzyme activity significantly decreased in the presence of silver nanoparticles and gold nanoclusters, most likely due to the lack of overlap between the emission spectrum and SPR peak. Our investigation showed that gold and silver ions significantly lowered the activity of luciferase. Released silver ions into the environment are highly reactive, inhibiting respiratory enzymes, increasing reactive oxygen species (ROS) production, binding sulfur- and phosphorus-containing molecules, interfering with cell defense systems, or exhausting intracellular concentrations of these molecules [41]. The emission spectra of firefly, *Renilla*, and mutant firefly luciferases were increased significantly in the presence of gold, gold–silver core–shell nanoparticles, and GNRs, respectively (Figure 3D). It may be suggested that the right position of metal nanoparticles and luciferase provides energy transfer and coupling. The energy transfer phenomenon between fluorescent chemicals and metal nanoparticles is well known. The absorption spectra of metal nanoparticles must be in the range of the luciferase bioluminescence spectra to cause bioluminescence energy transfer (560 nm for firefly luciferase and 480 nm for *Renilla* luciferase). This study demonstrated that the nanoparticles, with the most significant additive influence on the activity of luciferase, have an SPR peak in the emission spectrum range of the enzymes. Du et al. (2014) demonstrated that covalently linking gold nanocrystal (GNC) to horseradish peroxidase (HRP) significantly improved bioluminescence emission, while enhancement was only made possible when the GNC-HRP distance was within a restricted range of between 5–20 nm [6].

The SPR variations of noble metal nanoparticles are a well-established sensitive indication of biomolecule binding [42].

### 3.3. The Enzyme–Substrate Complexes Effect on SPR

**Figure 4:**
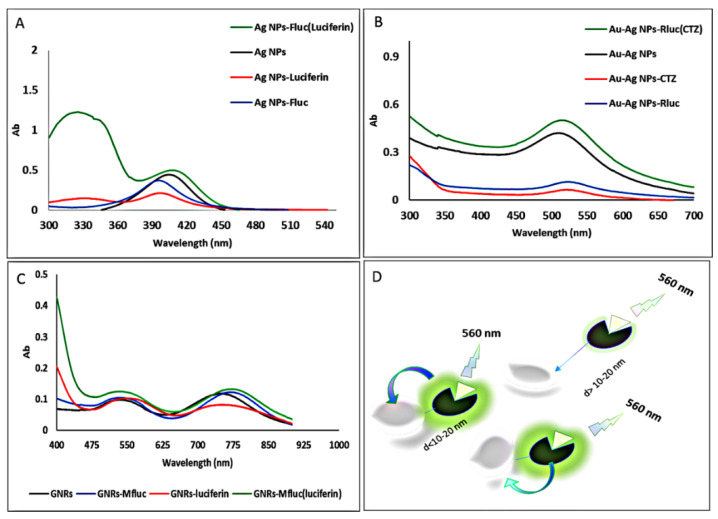
UV/visible absorption spectra of (**A**) Ag NPs, (**B**) Au–Ag NPs, and (**C**) GNRs with and without Rluc, Fluc, and Mfluc. (**D**) Schematic illustration of bioluminescence enhancement of luciferase–metal nanoparticles complex achieved through the coupling effect.

Complex formation between metal NPs and luciferase was monitored by comparing the UV/Vis spectra of metal NPs, enzymes, substrate, and enzyme–substrate mixtures (Figure 4A–C). As illustrated in Figure 1, all nanoparticles utilized in this investigation exhibited considerable absorption in the visible to infrared wavelength range. To examine the effect of enzymatic activity on changes in nanoparticle adsorption, we only chose nanoparticles with the most excellent additive impact on enzyme activity. The inclusion of enzymes reduced the metal NPs SPR peak and shifted the wavelength corresponding to its maximum, indicating the development of metal NP–luciferase enzyme complexes. Furthermore, we added only luciferin and coelenterazine to nanoparticles and observed a decrease in the nanoparticles’ absorption peak; when we added a mixture of enzyme and substrate to the nanoparticles, we observed an increase in the nanoparticles’ SPR peak intensity.

Adsorption of proteins on the metal nanoparticle surface is associated with several forces such as hydrogen bonds, solvation forces, and van der Waals interactions [43]. In addition, adsorption of a protein on the nanoparticle interface also depends on the affinity of the protein toward the nanoparticle surface and its ability to completely occupy the surface [17]. The surface of plasmonic nanoparticles is usually modified with electrolytes such as citrate or hexadecyl trimethyl ammonium bromide (CTAB) in order to improve their dispersibility. The surface modification provides enough net charges on the Au NPs, which may enable electrostatic attraction to the differently charged active groups in proteins [43]. Citrate-stabilized metal nanoparticles have carboxyl groups and negative charge on their surface. In addition to a carboxyl group, proteins have amine groups; hence, electrostatic and hydrogen forces are the main forces for the interaction of nanoparticles and proteins.

### 3.4. Nanoparticle Effect on Structural Properties

**Figure 5:**
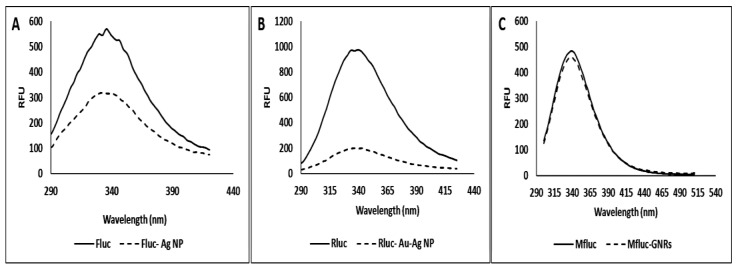
Tryptophan fluorescence spectra of enzymes (0.3, 0.6, and 0.8 µM for Fluc, Rluc, and Mfluc, respectively) in 50 mM Tris-HCl buffer and pH 7.8 in the present of (**A**) Silver nanoparticles (AgNPs), (**B**) Gold-Silver nanoparticles (Au-AgNPs) and (**C**) Gold nanorods (GNRs) respectively, and absence of metal NPs (0.01 nM).

The enzyme displays a fluorescence emission spectrum with a maximum at 338 nm (Figure 5) under our experimental conditions. This fluorescence emission maximum is characteristic of tryptophan (Trp) placed in a relatively hydrophobic environment [44,45]. When enzymes were immobilized on Ag, Au–Ag core–shell NPs, or GNRs, the fluorescence intensity dropped without shifting the maximum emission wavelength. The results revealed that metal NPs engaged and bonded with high affinity to enzymes, resulting in conformational alterations. The observed fluctuations in fluorescence intensity and maximum could result from the Trp environment changing, thereby altering the conformation of the enzyme. This alteration was associated with a new conformational state (unfolded state) [9]. This study showed that the addition of silver and gold–silver nanoparticles to firefly and *Renilla* luciferase reduced the fluorescence intensity of tryptophan, while gold nanorods had a minimal effect on the fluorescence intensity of mutant luciferase.

**Figure 6:**
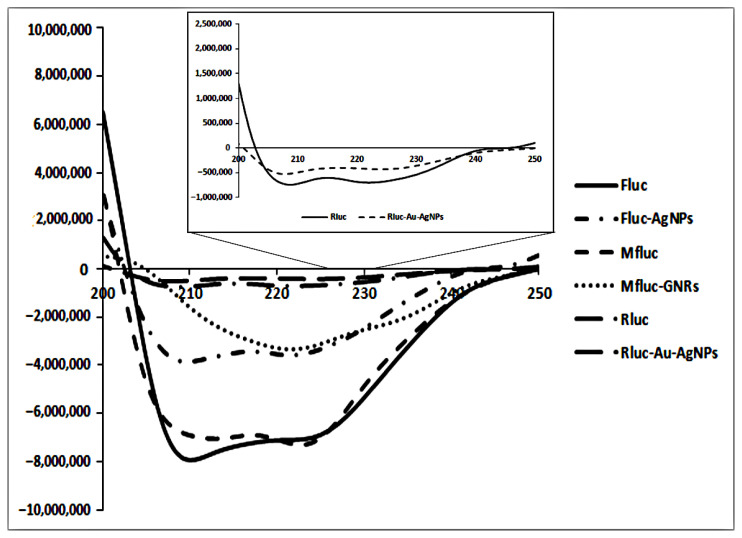
Far−UV CD spectra for luciferases with and without metal NPs.

The CD spectroscopy was performed to determine the influence of the noncovalent attachment of metal nanoparticles on the secondary structure of enzymes in Tris-HCl buffer (0.05 M, pH 7.8) at 25 °C (Figure 6). The far CD spectra of the luciferases with and without metal nanoparticles showed changes in their secondary structure. Results indicated firefly luciferase and its mutant form have almost the same secondary structure. In comparison to enzymes, the enzyme–nanoparticle complex showed a decrease in the α-helix contents of all luciferases (Table 1). As shown in Figure 6, Fluc and Mfluc had double negative peaks in the far CD spectra around 208 and 222 nm, while Rluc had two negative peaks in the range of 208 and 220 nm, but smaller than the other two enzymes (see inset in Figure 6). After incubating Fluc, Rluc, and Mfluc with metal NPs in Tris-HCl buffer (0.05 M, pH 7.8), we found a drop in α-helix secondary structure from 84% to 67%, 19% to 12%, and 47% to 43%, and a rise in beta-sheets from 1.24% to 3.24%, 31.52% to 38.78%, and 8.19% to 10.56% in Fluc, Mfluc, and Rluc, respectively. This result showed that these treatments caused structural changes in the secondary structures of luciferase; however, Au–Ag NPs affected the structure of Rluc less.

The metal nanoparticle size distribution and zeta potential were determined with and without luciferases using DLS (Table 2). The particle size distribution revealed that AuNPs, AgNPs, Au–AgNPs, BSA-AuNCs, and GNRs were polydispersed, with average diameter of ~29, 13.5, 3.0, 31, and 49.0 nm, respectively, and the corresponding average zeta potential values were −28, −24, −33, −39, and +15 mV, respectively. The increased metal nanoparticle size and a positive zeta potential value also confirmed the formation of the nanoparticle–enzyme complexes. The high negative potential value of the metal nanoparticles supports long-term stability, good colloidal nature, and high dispersity of metal nanoparticles due to the negative–negative repulsion and interaction with biomolecules.

### 3.5. AuNP Stability Test

**Figure 7:**
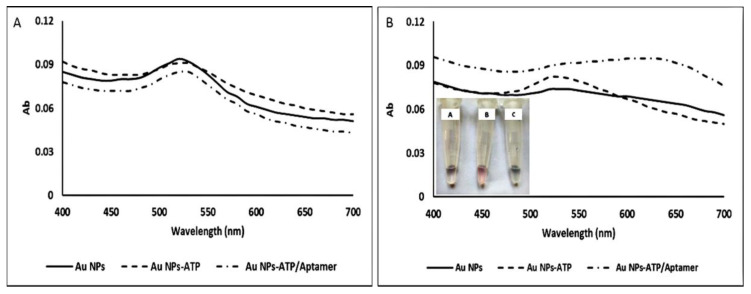
UV/Vis spectra of (**A**) AuNPs, the AuNPs mixed with 2 mM ATP, and the AuNPs mixed 2 mM ATP and 5 μM aptamer after 1 min incubation; (**B**) AuNPs and others mixed with 100 mM NaCl (inset). (**A**) AuNPs, (**B**) AuNPs/ATP, (**C**) AuNPs/ATP–aptamer citrate–AuNPs, and AuNPs/ATP–aptamer turned blue with 100 mM NaCl, but ATP stabilized the AuNPs because of its negative charges.

In this study, we investigated the effect of the target molecules and the aptamer bound to the target molecules on the stability of nanoparticles and luciferase activity. Figure 7 shows that gold nanoparticles were aggregated in the presence of 100 mM sodium chloride salt, and a blue color change was observed. In addition, although single-stranded DNA molecules make nanoparticles stable, they cannot protect nanoparticles from aggregation if they bind to the target molecule. While ATP has multiple negative charges and can stabilize the AuNPs in the presence of salt [37], changes in the stability of gold nanoparticles in the presence and absence of the target molecule can be seen through absorption spectroscopy.

### 3.6. ATP Assay

**Figure 8:**
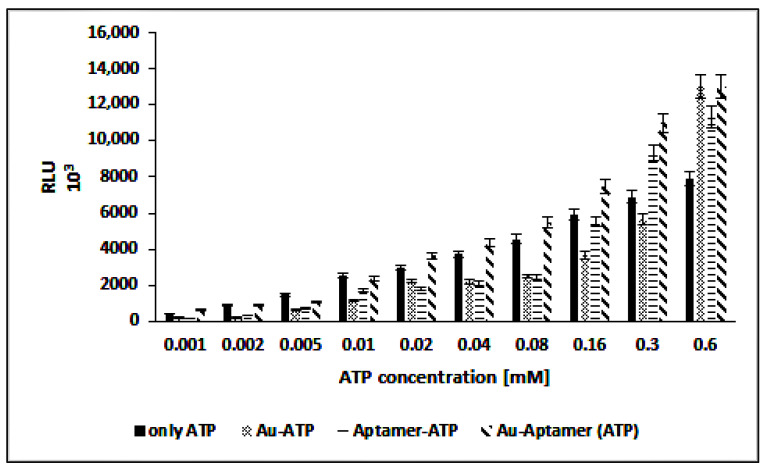
Luciferase activity in the presence of ATP stabilized AuNPs, aptamer–ATP and aptamer–ATP adsorbed on the AuNPs pre-incubated with different concentrations of ATP.

The efficiency of the nanoparticles, aptamer, and complex of aptamer and nanoparticles was determined for detecting different concentrations of ATP (1–600 µM) in the presence of firefly luciferase. The results in Figure 8 shows that, in the presence of nanoparticles, aptamer, and the complex of aptamer and nanoparticles, the luciferase activity was higher than only ATP in all tested ATP concentrations. 

Furthermore, in addition to binding to the aptamer, ATP is easily attached to the surface of the nanoparticles, increasing its availability for the luciferase enzyme. The effect of gold nanoparticles on increasing luciferase activity was also mentioned in the previous sections. The bioluminescence-based assay for ATP is a sensitive method, but the intensity of the bioluminescence signal is usually low for low concentrations. The results of this study show that the presence of gold nanoparticles increases the intensity of the bioluminescence signal. It is an advantage to measure the low concentrations of the analyte, especially ATP, and the presence of aptamer increases the selectivity. The results in Figure 8 show that the signal intensity in the presence of gold nanoparticle–ATP complexes was higher than only ATP in concentrations of 5–40 μM.

### 3.7. Cell Culture

**Figure 9:**
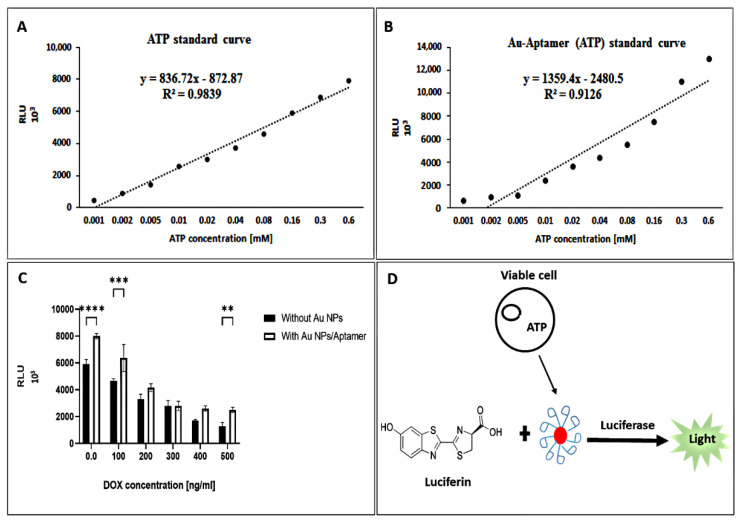
(**A**,**B**) Linear detection range of ATP (**C**) Bioluminescence detection of ATP in MCF−7 cell culture with DOX different concentration. (**D**) Schematic illustration of cell viability assay by ATP bioluminescence assay. ** *p* values < 0.01, *** *p* values < 0.001, **** *p* values < 0.0001.

Bioluminescent ATP assays take advantage of the firefly luciferase enzymatic reaction, which uses ATP from viable cells to generate photons of light. Viable cells were lysed to release the ATP for detection, and reagents containing firefly luciferase and substrate were added to catalyze a two-step reaction. Figure 9 displays a good linear relationship between the bioluminescence intensity of the luciferase and the added ATP concentration. The bioluminescence intensity increased with the ATP concentration (1–600 µM) with 5 µM as the limit of detection (LOD) and 160 µM as the limit of quantitation (LOQ) for ATP.

To further confirm that the assay is also applicable to ATP detection, we conducted the methodology to quantify ATP in MCF-7 cell extracts treated with different concentrations of doxorubicin. Figure 9C shows the luciferase activity with and without the AuNP/aptamer colloid in the presence of cell lysates treated with DOX concentrations of 0, 100, 200, 300, 400, and 500 ng·mL^−1^. We estimate that the concentration of ATP in the cell lysate was 1.8–5.8 ± 0.06 mM; this value agrees with the normal range of ATP between 2 and 10 mM in cells [28,46]. The bioluminescence is proportional to the ATP concentration when ATP is the limiting component in the luciferase reaction. A higher luminescent signal indicates higher ATP levels. A comparison of this study with other ATP detecting methods is summarized in Table 3.

## 4. Discussion

Luciferases were considered in this study due to their roles in chemical transformations leading to light emission with many biological applications, including beneficial reporter proteins for gene expression, ATP assays, environmental bioassays based on the changes in bioluminescent intensities, and biosensors. In summary, according to the results presented in this communication, citrate-stabilized AuNPs, AgNPs, Au–AgNPs, BSA-AuNCs, and GNRs nanoparticles had diameters of ~29, 13.5, 3.0, 31, and 49.0 nm, respectively, and exhibited typical absorption bands. Electrostatic interactions, dynamic exchange with citrate, and hydrogen bonding to form a protein corona demonstrate the enzyme’s attachment to the nanoparticle surface, as reported earlier [52]. CD and fluorescence spectroscopies were used to study the enzyme–nanoparticle interaction. We detected a significant drop and change in the ellipticity and emission maximum. We effectively demonstrated that all-metal nanoparticles and gold nanorods boosted the activity of firefly and *Renilla* luciferase by five times compared to free enzymes after incubation at room temperature. However, mutant luciferase activity significantly decreased in the presence of silver nanoparticles and BSA–Au nanoclusters. Käkinen et al. (2013) reported a dose-dependent inhibition of firefly luciferase activity boosted by citrate-coated Ag nanoparticles (AgNPs). The reaction of functional groups of proteins, especially S– and N– with Ag+ ions released by the metal NPs stimulated enzyme inhibition rather than protein unfolding [41].

The results indicated that nanoparticles with an SPR peak that overlaps with the emission spectrum of enzymes have a more significant additive effect. Additionally, we discovered an additive influence of metal nanoparticles on the emission spectra of enzymes. The nanoparticles showed an additive effect on immobilized enzymes. Furthermore, five distinct physicochemical mechanisms contribute to the enhancement of enzyme activity in the nanoparticle–enzyme complex: increased enzyme density on the surface of the nanoparticles; facilitated substrate mass transfer due to the high surface-to-volume ratio of nanoparticles, which absorbed both the substrate and the enzyme; nanoparticle morphology; nanoparticle surface chemistry; optimal enzyme orientation for enhanced enzyme–substrate interactions [52].

This study showed that, similar to the phenomena of fluorescence and chemiluminescence, bioluminescence could be coupled with the surface plasmon of metal nanoparticles, as reported earlier [20]. Previously published research indicates that luminescence enhancement can also occur due to catalysis (as in chemiluminescence) or plasmon-coupled enhancement [53]. We investigated the effect of the enzymatic reaction and light emission on the absorption spectrum of nanoparticles. The results indicate that combining luciferase–substrate enzyme and light emission that overlaps with the SPR peak of nanoparticles increases the adsorption of nanoparticles at the maximum absorption wavelength compared to nanoparticles alone or nanoparticle–enzyme and nanoparticle–substrate complexes. In metal-enhanced fluorescence (MEF), fluorescent species are stimulated by an external light source. The energy emitted by the electronically excited states (fluorescence emission) is partially transferred (coupled) to surface plasmons in metals due to the excitation. This phenomenon is referred to as an “induced mirror dipole in the metal” in some circles [15,16]. In summary, we carefully dissected the target, aptamer, and AuNP interactions using ATP as the model target. We designed a bioluminescence-based aptasensor for ATP detection and used gold nanoparticles to increase the availability of ATP and increase the intensity of the bioluminescence signal. The results showed that, in the presence of ATP nanoparticles and aptamer, luciferase activity was higher compared to only ATP, nanoparticles, and aptamer. In addition, to investigate the effectiveness of designed ATP sensor, doxorubicin was used as a common cancer treatment drug on MCF-7 cells at different concentrations. There is a good linear relationship between the bioluminescence intensity of the luciferase and the added ATP concentration. The bioluminescence intensity increased with the ATP concentration (1–600 µM). The ATP bioluminescence method has advantages including the direct detection of ATP in aqueous solution and cell extract without the need for separation and high-cost equipment, fast and easy operation, rapid feedback, and practical maneuverability. However, the stability and activity of luciferase still need improvement [54,55]. Therefore, it may be concluded that the addition of metal NPs similar to some additive or point mutations can increase the sensitivity and activity of firefly luciferase toward ATP, making it a suitable sensor for cell viability as reported earlier [56,57,58,59,60,61,62,63,64,65].

## Figures and Tables

**Table 1:** Determination of α-helix and β-strand relative content (%). (http://cbdm-01.zdv.uni-mainz.de/~andrade/k2d2/, accessed on 2 September 2022).

Samples	α-Helix (%)	β-Strand (%)
Fluc	84.27	1.24
Fluc-AgNPs	67.45	3.24
Rluc	47.93	8.19
Rluc-Au-AgNPs	43.94	10.56
Mfluc	19.69	31.52
Mfluc-GNRs	12.96	38.78

**Table 2:** Particle size distribution of metal NPs with and without luciferases.

	Size				Zeta			
	NPs	Rluc	Fluc	Mfluc	NPs	Rluc	Fluc	Mfluc
Au NPs	29.0	183.0	290.0	72.0	−28.0	+13.0	+6.0	−7.0
Ag NPs	13.5	82.0	44.0	153.3	−24.0	−9.0	−6.0	−8.0
Au-Ag NPs	3.0	55.0	412.0	80.12	−33.0	−9.0	−6.0	−9.0
BSA-Au NCs	31.0	119.0	98.0	52.04	−39.0	−12.0	−4.0	−6.0
GNRs	49.0	589.0	400	1529.0	+15.0	−1.0	+2.0	−5.0
Enzymes	-	68.0	61.0	69.0	-	−2.0	−9.0	−4.0

**Table 3:** Comparison of some methods used for ATP detection.

Methods/Materials Used	Analytical Ranges/LODs (μM)	Samples	References
Luminescence/luciferase, luciferin	5 nM–100 μM	Cell extract	[47]
Luminescence/hexokinase, glucose, luminol	0.30–80/70	pharmaceuticals, milk, and soils	[48]
Luminescence/aptamer, [Ru(phen)2(dppz)]2+	1–100 nM/1 nM	-	[49]
Luminescence/aptamer, peroxidase, luminol	1–10/0.1	Cancer cell extract	[50]
Chemiluminescence/luminol-H_2_O_2_- HRP-fluorescein	1–10/0.32	Cancer cell extract	[50]
Fluorescence polarization/QD-DNA	10–350/3.7	Serum	[51]
**Bioluminescent/luciferase, luciferin, aptamer, Au NPs**	1–600/5	Cancer cell extract	This work

## Data Availability

Not applicable.
